# Glutathione metabolism rewiring protects renal tubule cells against cisplatin-induced apoptosis and ferroptosis

**DOI:** 10.1080/13510002.2022.2152607

**Published:** 2023-01-24

**Authors:** Xing-Qiang Dong, Li-Kai Chu, Xu Cao, Qian-Wei Xiong, Yi-Ming Mao, Ching-Hsien Chen, Yun-Li Bi, Jun Liu, Xiang-Ming Yan

**Affiliations:** a Department of Urology, Children’s Hospital of Soochow University, Suzhou, People’s Republic of China; b Department of Thoracic Surgery, Suzhou Kowloon Hospital, Shanghai Jiao Tong University School of Medicine, Suzhou, People’s Republic of China; c Department of Internal Medicine, Division of Nephrology, University of California Davis, Davis, CA, USA; d Department of Internal Medicine, Division of Pulmonary and Critical Care Medicine, University of California Davis, Davis, CA, USA; e Pediatric Institute of Soochow University, Children’s Hospital of Soochow University, Suzhou, People’s Republic of China

**Keywords:** Glutathione metabolism, renal tubular, apoptosis, ferroptosis, JAK/STAT3, Cisplatin, Baicalein, Acute kidney injury

## Abstract

Renal proximal tubular cells are highly vulnerable to different types of assaults during filtration and reabsorption, leading to acute renal dysfunction and eventual chronic kidney diseases (CKD). The chemotherapeutic drug cisplatin elicits cytotoxicity causing renal tubular cell death, but its executing mechanisms of action are versatile and elusive. Here, we show that cisplatin induces renal tubular cell apoptosis and ferroptosis by disrupting glutathione (GSH) metabolism. Upon cisplatin treatment, GSH metabolism is impaired leading to GSH depletion as well as the execution of mitochondria-mediated apoptosis and lipid oxidation-related ferroptosis through activating IL6/JAK/STAT3 signaling. Inhibition of JAK/STAT3 signaling reversed cell apoptosis and ferroptosis in response to cisplatin induction. Using a cisplatin-induced acute kidney injury (CAKI) mouse model, we found that inhibition of JAK/STAT3 significantly mitigates cisplatin nephrotoxicity with a reduced level of serum BUN and creatinine as well as proximal tubular distortion. In addition, the GSH booster baicalein also reclaims cisplatin-induced renal tubular cell apoptosis and ferroptosis as well as the *in vivo* nephrotoxicity. In conclusion, cisplatin disrupts glutathione metabolism, leading to renal tubular cell apoptosis and ferroptosis. Rewiring glutathione metabolism represents a promising strategy for combating cisplatin nephrotoxicity.

## Introduction

Acute kidney injury (AKI), featured by the rapid decline in renal function, is caused by a broad range of etiologies such as ischemia and reperfusion (IR), sepsis, and nephrotoxic drugs [1]. AKI impacts more than 10 million patients with approximate mortality of 1.7 million deaths per year worldwide [2]. Among that, 8%-60% of the total AKI cases are associated with the nephrotoxic chemotherapeutic drug, cisplatin [3]. Moreover, the incidence of cisplatin-induced AKI has been reported to occur in up to 46% of patients treated with high-dose cisplatin [4]. Nevertheless, effective therapeutic strategies for AKI are yet available owing to the paucity of understanding of the precise molecular mechanisms.

The kidney is responsible for the metabolism and excretion of cisplatin in the body, thus acquiring the disproportionate accumulation of cisplatin in renal proximal tubular cells, making the kidney one of the most susceptible organs to cisplatin toxicity [5]. The nephrotoxicity of cisplatin is largely manifested by the loss of renal proximal tubular cells in an apoptotic way, leading to an irreversible transition of renal function. The apoptosis-targeted remedy has been tested but failed to completely combat cisplatin nephrotoxicity [6–8], suggesting the existence of a previously unanticipated mechanism of action that underpins cisplatin-induced AKI.

Ferroptosis, a type of programmed cell death that is genetically and biochemically distinct from apoptosis, can be triggered by multiple types of stimuli and characterized by the accumulation of lipid peroxides [9]. Ferroptosis is also observed in a variety of cancer types upon cisplatin treatment and acts as a different mechanism of cell death for cancer therapy [10–12]. Ferroptosis is closely linked to the nephrotoxicity of cisplatin as they could induce mitochondria oxidative stress through disrupting antioxidation molecular and enzymes such as glutathione (GSH) and glutathione peroxidase 4 (GPX4), leading to the accumulation of excessive reactive oxygen species (ROS), which activates iron-dependent renal tubular cells ferroptosis [13]. Moreover, a distinct type of cell death, necroptosis, occurs during cisplatin-induced AKI [14,15]. Therefore, elucidating the precise mechanism of action for cisplatin-related nephrotoxicity is desired to develop interventions for renal function protection from AKI.

Besides the formation of cisplatin-DNA adducts to induce cell death, the toxicity of cisplatin is also displayed by inducing oxidative stress. Under physiological conditions, the intracellular reactive oxygen species levels are eliminated by the scavenging system including GSH. As one of the most abundant thiols, GSH is synthesized exclusively in the cytosol by the action of enzymes including glutamate-cysteine ligase (GCL) and glutathione synthetase (GSS), and is transported into mitochondria for the action of antioxidation [16]. In addition, the intracellular balance of GSH is maintained by several antioxidative enzymes including glutathione-disulfide reductase (GSR) and glutathione peroxidase 4 (GPX4) [17]. Disruption of GSH metabolism renders cell organelles vulnerable to stress-induced damage, leading to cell death, tissue remodeling as well as the development of diseases. In renal tubular cells, GSH is depleted under cisplatin induction leading to mitochondria oxidative stress-mediated cell apoptosis, renal dysfunction, and GPX4-mediated lipid peroxidation-related ferroptosis. Direct application of antioxidative drugs displayed limited renal function regression on cisplatin nephrotoxicity.

Here, we hypothesized that both apoptosis and ferroptosis were triggered in renal tubular cells in response to cisplatin. we aimed to investigate the mechanism of action underpinning cisplatin-induced nephrotoxicity. We found cisplatin-induced renal proximal tubular cells both apoptosis and ferroptosis by disrupting GSH metabolism. We identified that IL6/JAK/STAT3 signaling was activated and aggravated cell apoptosis and ferroptosis upon cisplatin induction. Direct supplement of GSH or IL6/JAK/STAT3 signaling blockade attenuated renal tubular cells apoptosis and ferroptosis in vitro as well as relieving renal function in vivo in response to cisplatin treatment that represents a promising therapeutic strategy against cisplatin nephrotoxicity.

## Materials and methods

### Reagents and cell culture

Z-VAD-FMK (S7023, Selleck), ferrostatin-1 (S7243, Selleck), NAC (S1623, Selleck), and ruxolitinib (S1378, Selleck) were purchased from Selleck Chemicals LLC (Houston, TX, USA). Normal human proximal tubular cell line HK2 were from the American Type Culture Collection (ATCC) and cultured in DMDM/F12 medium (Gibco) supplemented with 10% Fetal Bovine Serum (FBS, Gibco) and 1% penicillin/streptomycin (Thermo Fisher Scientific) and maintained in a humidified incubator with 5% CO_2_ at 37°C.

### Cell viability

HK2 cells were seeded in a 96-well plate with 8000 cells per well and incubated overnight. Then, cells were treated with different doses of cisplatin (S1166, Selleck) or baicalein (S2268, Selleck) as indicated for 24, 48, or 72 h. After that, the medium was removed and replaced by a fresh medium containing CCK8 reagent (K1018, APExBIO, TX, US). The plate was incubated for 1 h and OD450 was measured by microplate reader MultiskanTM FC (Thermo Fisher Scientific).

### Western blot

Total proteins were isolated from frozen kidney tissue or cultured cells using RIPA lysis buffer (R0010, Solarbio, Beijing, China) containing protease and phosphatase inhibitor cocktail (04693116001, Roche). Protein samples were separated in a 10% SDS page gel and transferred onto the PVDF membrane (R1SB07718, Millipore). After the blocking using 5% skimmed milk for 1 h at room temperature and three times washing with 1×PBST, the membrane was incubated with primary antibodies at 4°C overnight and then with the corresponding secondary antibodies with a dilution of 1:4000 for 1 h at room temperature. Proteins were visualized by enhanced chemiluminescence (ECL, 32209, Thermo Fisher Scientific). Primary antibodies against β-actin (4970S, Cell Signaling, 1:1000 dilution), GPX4 (ab125066, Abcam, 1:1000 dilution), GSS (SC-166882, Santa Cruz, 1:500 dilution), GSR (SC-133245, Santa Cruz, 1:500 dilution), JAK2 (3230S, Cell Signaling, 1:1000 dilution), phospho-JAK2 (3771S, Cell Signaling, 1:500 dilution), STAT3 (9139S, Cell Signaling, 1:1000 dilution), phospho-STAT3 (9145S, Cell Signaling, 1:1000 dilution). Secondary antibodies against anti-mouse HRP-linked antibody (7076S, Cell Signaling, 1:5000 dilution), anti-rabbit HRP-linked antibody (7074S, Cell Signaling, 1:5000 dilution).

### Mouse model of cisplatin-induced AKI and drug administration

Six to eight-week-old C57BL/6J mice were purchased from the Jackson Laboratory and fed food and water ad libitum. Mice were intraperitoneally injected with one dose of cisplatin (S1166, Selleck) at 30 mg/kg or the same amount of saline for control. At 72 h after administration, the mice were sacrificed, the tissues were collected for histological analysis, and blood serum was collected for assaying the creatinine and BUN levels. For drug administration, mice were intraperitoneally injected with one dose of 5 mg/kg of the STAT3 inhibitor S3I-201 (S1155, Selleck) or 10 mg/kg anti-apoptotic and anti-ferroptotic compound baicalein (S2268, Selleck) at the opposite flank after cisplatin administration. All mouse experiments were approved by the Institutional Animal Care and Use Committee (IACUC) of The Children’s Hospital of Soochow University.

### Flow cytometry

For analysis of cell apoptosis, single-cell suspension of HK2 was washed with cold PBS and adjusted cell number to 1×10^6^. The cells were resuspended in 100μL of PBS containing 5 μL Annexin V-FITC and 10 μL PI (556547, BD Biosciences) and incubated for 30 min at room temperature; for analysis of lipid peroxidation-related ferroptosis, single cells suspension of HK2 were stained with Liperfluo (L248, Dojindo, Kumamoto, Japan), a fluorescence probe for lipid peroxide detection,in PBS containing 2% (w/v) bovine serum albumin (BSA) on ice for 30 min according to the manuscript’s instruction. The relative ROS concentration was assessed using DCFH-DA (S0033S, Beyotime) according to the manufacturer’s instructions. The ROS level was detected using flow cytometry at an excitation wavelength of 488 nm and an emission wavelength of 525 nm. The fluorescent signals were acquired using a Beck-man Coulter Gallios Flow Cytometer (BECKMAN COULTER) and analysed using FlowJo software (v10.6).

### Histopathological analysis and immunohistochemistry

The paraffin-embedded slides (3–5 μm) containing kidney tissue were prepared and stained with HE reagent, according to standard protocol. Stained sections were analyzed using a light microscope (Zeiss). For IHC staining, tissue slides were deparaffinized in xylene and rehydrated in alcohol and followed by a serial procedure of deactivation of endogenous peroxidase and antigen retrieval with 3% H_2_O_2_ and 0.1 M sodium citrate (pH 6.0), respectively. Subsequently, the slides were blocked with 5% normal goat serum and probed with primary antibodies anti-4HNE (ab48506, Abcam, 1:50 dilution) or cleaved caspase 3 (9964S, Cell Signaling, 1:200 dilution), followed by the incubation with the corresponding secondary antibodies, after which slides were counterstained with hematoxylin to visualize nuclei. Images were acquired with a light microscope (Zeiss).

### GSH, GSH/GSSG ratio, and IL6 level determination

The total GSH content and GSH/GSSG ratio in the renal cortex, which was collected by excision across the outer stripe of the outer medulla under an anatomical microscope (Olympus) according to the previous study [18], was determined using GSH and GSSG Assay Kit (S0053, Beyotime) according to the manufacturer’s instructions. The OD value was read by using a microplate reader MultiskanTM FC (Thermo Fisher Scientific) at a wavelength of 412 nm and normalized to the control group. The IL6 level was detected in the culture media using the IL6 ELISA Assay kit (SEKH-0013, Solarbio) according to the manufacturer’s instructions.

### Gene set enrichment analysis

Transcriptomic datasets (GSE106993) for the kidneys from control or cisplatin-treated mouse were used in the present study. The transcript per million (TPMs) were used for normalization and the differentially expressed genes (DEGs) were analysed using the R package ‘limma’ (v.4.1). Gene set enrichment analysis (GSEA) was performed using the gene sets of C2 KEGG and Hallmark from the Molecular Signatures Database (https://www.broadinstitute.org/gsea/msigdb/).

### Statistical analysis

Statistical analyses were performed using Prism 8 (GraphPad Software, San Diego, CA, USA). *P* values were calculated by two-tailed unpaired Student’s t-test between two groups. A regression analysis was performed if applicable. A *p* < 0.05 was considered significant.

## Results

### Cisplatin induces dysregulation of GSH metabolism in the renal tubules

**Figure 1.**
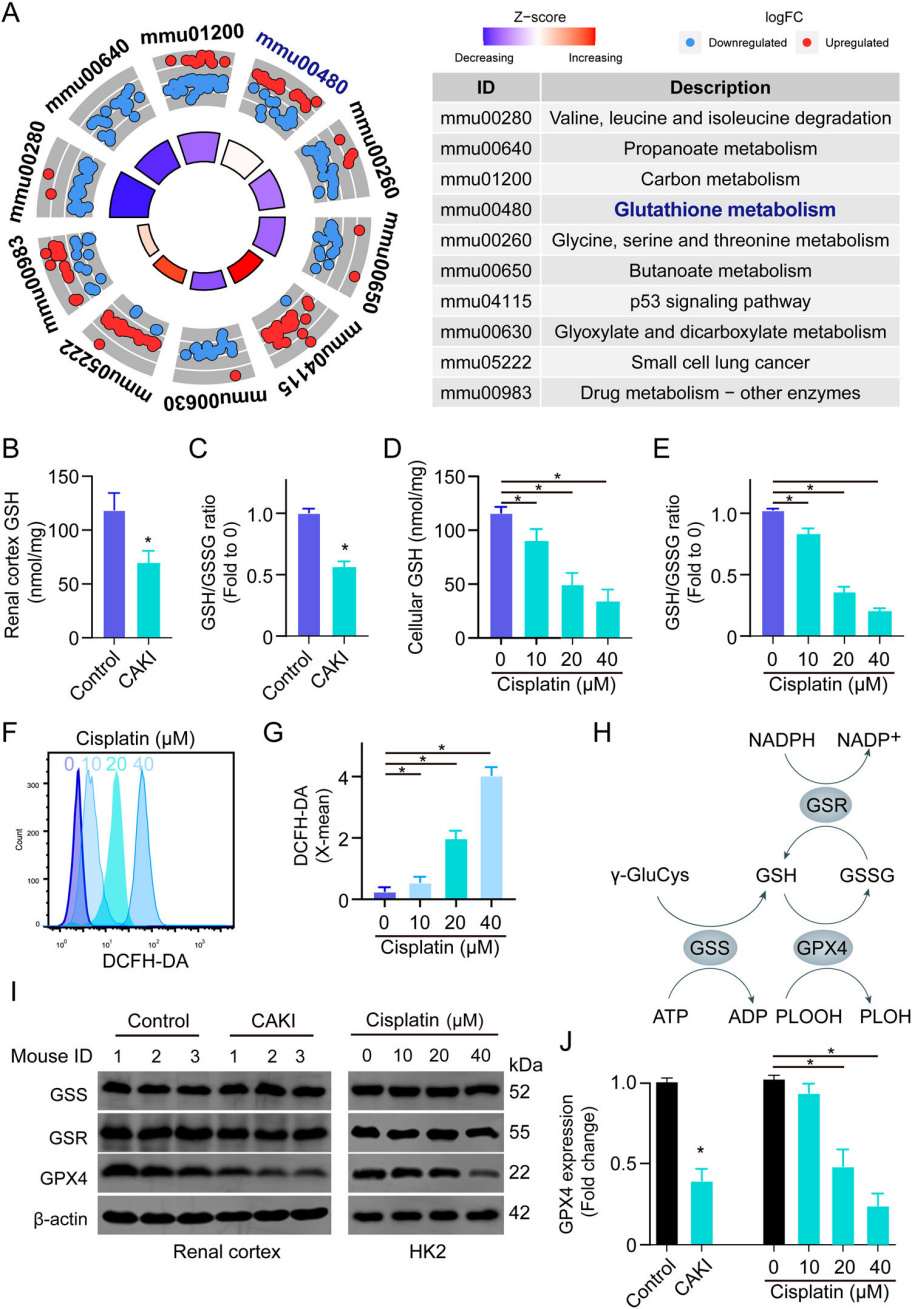
Cisplatin induces dysregulation of GSH metabolism in the renal tubules. (A) The enriched signaling pathways in cisplatin-induced kidneys using KEGG analysis, mmu: Mus musculus. (B, C) The total GSH level and GSH/GSSG ratio in the renal cortex of kidneys from saline (Control) or cisplatin-induced acute kidney injury mice (CAKI). (D, E) The total GSH and GSH/GSSG ratio levels in HK2 cells treated with different doses of cisplatin. Data are presented as mean ± S.E.M., *p<0.05. (F, G) The ROS level and quantification in HK2 cells treated with different doses of cisplatin. (H) Schematic illustration showing the GSH metabolism pathway. (I, J) The protein expressions of GSS, GSR, and GPX4 in the renal cortex of the kidneys from saline or cisplatin-treated C57BL/6J mouse (*n* = 3 per group) or in the cell lysate of PBS or cisplatin-treated HK2 cells using immunoblot. Statistical significance between the two groups as indicated was determined using an unpaired two-tailed Student’s t-test. The experiments were repeated at least in triplicate.

To investigate the mechanism of action underlying cisplatin-induced nephrotoxicity, we interrogated the RNA-seq data of the kidneys from control and cisplatin-treated mice (GSE106993). KEGG analysis revealed that the enriched genes were associated with signaling pathways including amino acid degradation and metabolism and p53 signaling, suggesting cisplatin imposed metabolic changes on the kidney (Figure 1A). Among that, glutathione (GSH) metabolism is one of the most dysregulated signaling pathways and is linked to mitochondria oxidative phosphorylation in multiple cellular processes [19]. We next focused our attention on GSH metabolism and found that GSH metabolism was dysregulated with a significantly decreased level of total GSH and GSH/GSSG ratio in the renal cortex in response to cisplatin treatment (Figure 1B, 1C). Moreover, increased ROS, decreased total GSH levels, and GSH/GSSG ratio in renal tubular cells were induced by cisplatin treatment dose-dependently in vitro (Figure 1D-1G). GSH synthesis is orchestrated by intracellular enzymatic machinery including glutathione synthetase (GSS), glutathione-disulfide reductase (GSR), and the ferroptosis regulator glutathione peroxidase 4 (GPX4) (Figure 1H). Accordingly, the protein level of GSS, GSR, and GPX4 were investigated and showed significantly reduced levels of GPX4 in renal tubular cells and the renal cortex in response to cisplatin induction, whereas cisplatin treatment had minimal impact on the expression of GSS and GSR (Figure 1I, 1J). The data indicates cisplatin impairs GSH metabolism in renal tubular cells.

### Cisplatin induces renal tubule cells both apoptosis and ferroptosis

**Figure 2.**
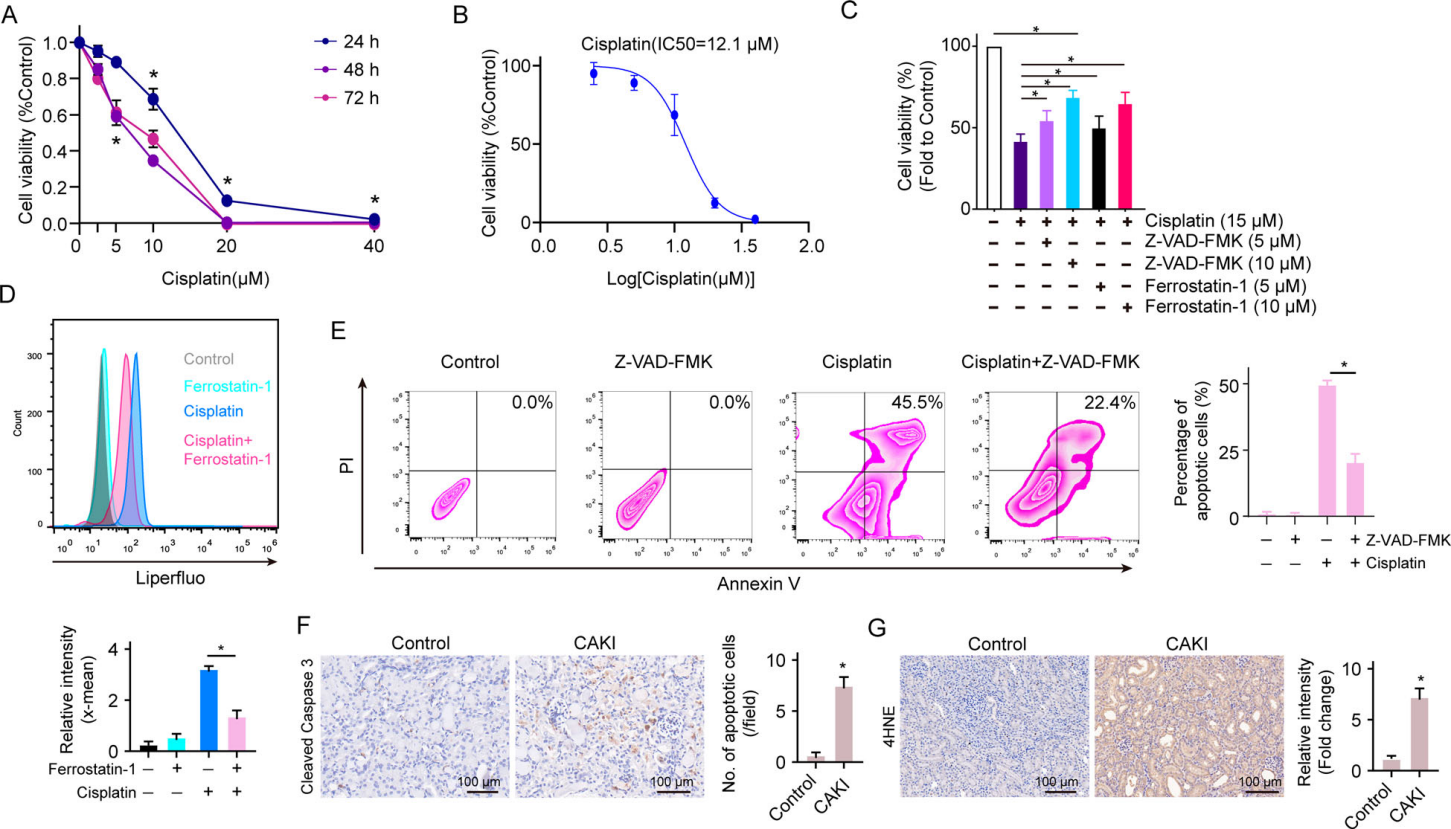
Cisplatin induces renal tubule cell both apoptosis and ferroptosis. (A) Cell viability was detected in HK2 cells treated with different doses of cisplatin for 24, 48, and 72 h. (B) IC50 of cisplatin on HK2 cells at the time point of 24 h was determined. (C) Cell viability was detected in HK2 cells treated with 10 μM ferrostatin-1 or Z-VAD-FMK in the presence or absence of 15 μM of cisplatin for 24 h. (D) Cell ferroptosis were analysed in HK2 cells treated with 15 μM of cisplatin with the addition of 10 μM ferrostatin-1. (E) Cell apoptosis was analysed in HK2 cells treated with 15 μM of cisplatin with the addition of 10 μM Z-VAD-FMK. The experiments were at least in triplicate. IHC staining of apoptotic marker cleaved caspase 3 (F) and ferroptosis marker 4HNE (G) on the kidney from C57BL/6J mice treated with or without 30 mg/kg cisplatin for 72 h. Data are mean ± S.E.M. Statistical significance between the two groups as indicated was determined using an unpaired two-tailed Student’s t-test, *n *= 5, *p<0.05.

GSH depletion leads to the accumulation of intracellular ROS, which is linked to cell apoptosis and ferroptosis, two main types of cell death in renal tubular cells [20]. We investigated the response of renal tubular cells to cisplatin and found that, after cisplatin induction, renal tubular cells displayed reduced cell viability dose-dependently with an IC50 of cisplatin is 12.1 μM at 24 h (Figure 2A, 2B). To investigate if apoptosis and ferroptosis occurred simultaneously in renal tubule cells in response to cisplatin induction, the apoptosis inhibitor Z-VAD-FMK and ferroptosis inhibitor ferrostatin-1 were used to treat renal tubule cells in the presence of cisplatin. We found both Z-VAD-FMK and ferrostatin-1 could effectively attenuate the cytotoxicity of cisplatin (Figure 2C). Moreover, the apoptotic and ferroptotic features induced by cisplatin were significantly reversed by Z-VAD-FMK and ferrostatin-1, respectively (Figure 2D, 2E). By using a mouse model of cisplatin-induced acute kidney injury (CAKI) according to our previous study [21], we also found the kidney of cisplatin-induced mice displayed apparent apoptotic and ferroptotic features with significantly increased levels of cleaved caspase-3 and 4HNE expression (Figure 2F, 2G), respectively. The data show cisplatin induces renal tubule cells both apoptosis and ferroptosis simultaneously.

### Cisplatin induces activation of IL6/JAK/STAT3 signaling

**Figure 3.**
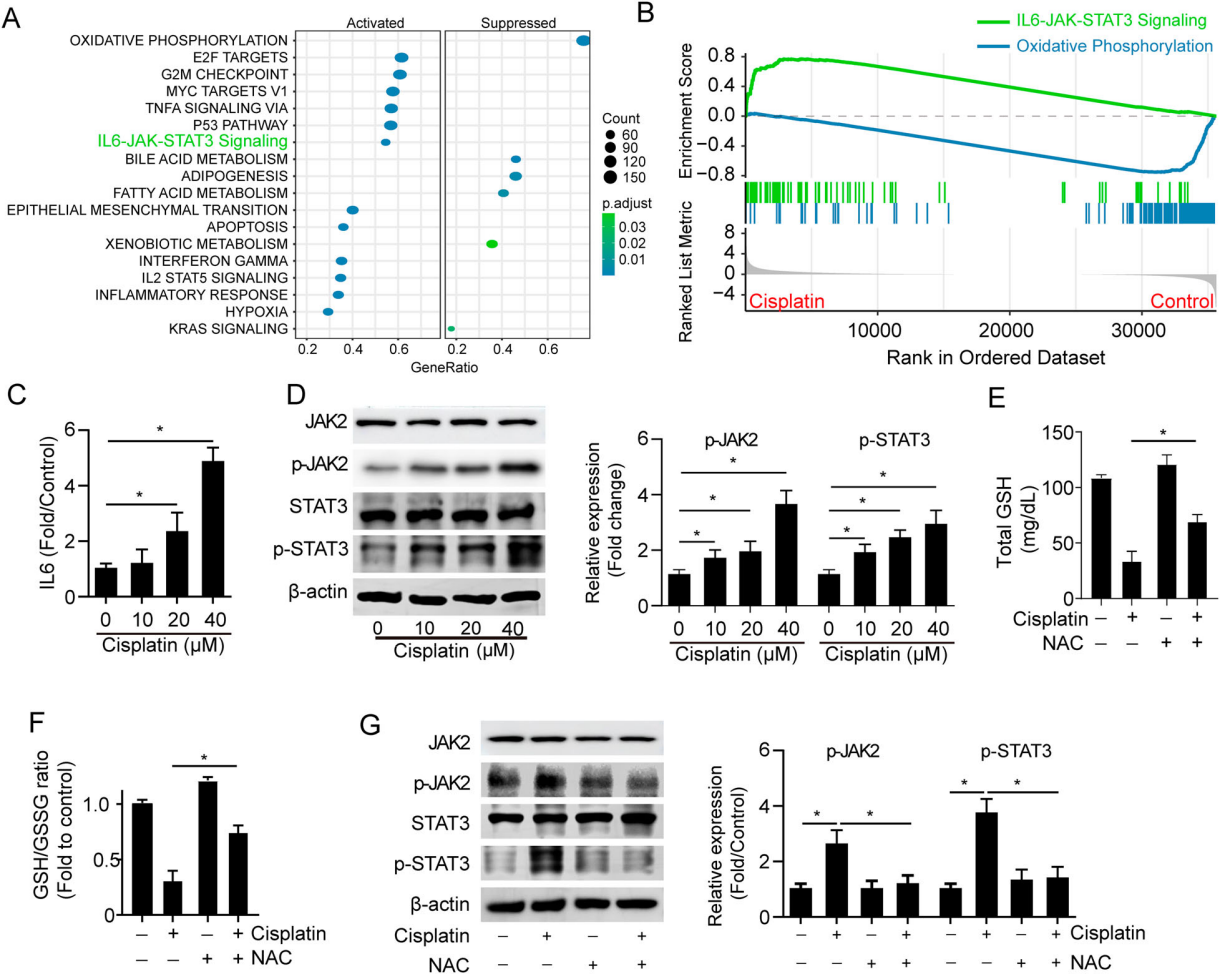
Cisplatin induces activation of IL6/JAK/STAT3 signaling. (A) GSEA analysis showed the enriched signaling pathways in the cisplatin-induced mouse kidney. (B) IL6-JAK-STAT3 signaling was upregulated and oxidative phosphorylation was downregulated in cisplatin-induced mouse kidneys using GSEA analysis. (C) The total IL6 level in HK2 cells was treated with different doses of cisplatin (0-40 μM) for 24 h. (D) The expressions of STAT3 and phospho-STAT3 (p-STAT3) in HK2 cells were treated with different doses of cisplatin (0-40 μM) for 24 h. (E-G) The total GSH level (E) and GSH/GSSG ratio (F) expression of JAK2, p-JAK2, STAT3, and p-STAT3 (G) in HK2 cells treated with 15 μM cisplatin in the presence or absence of 10 μM NAC. The experiments were repeated at least in triplicate. Data are presented as mean ± S.E.M. Statistical significance between the two groups as indicated was determined using an unpaired two-tailed Student’s t-test, *p<0.05.

We next investigated the signaling pathways controlling both apoptosis and ferroptosis in response to cisplatin by performing GSEA analysis on the RNA sequencing data of cisplatin-treated mouse kidneys and found an enrichment of genes that are known to participate in oxidation phosphorylation, cell cycle, p53 signaling as well as inflammatory responses in cisplatin-treated mouse kidneys (Figure 3A). In addition, the oxidation phosphorylation was impaired as expectedly (Figure 3B, blue line); and IL6/JAK/STAT3 signaling was activated under cisplatin treatment (Figure 3B, green line). As IL6/JAK/STAT3 signaling was negatively regulated by GSH and associated with cell apoptosis [22], we assumed IL6/JAK/STAT3 played a pivotal role in renal tubular cell apoptosis and ferroptosis upon cisplatin induction. Expectedly, the level of inflammatory cytokine IL6 was significantly increased by cisplatin treatment (Figure 3C). We also found the JAK/STAT3 signaling was upregulated upon cisplatin treatment (Figure 3D). Next, we investigated whether JAK/STAT3 activation triggered by cisplatin was through GSH depletion. We found N-acetyl cysteine (NAC), an antioxidant used to directly replete GSH levels, significantly boosted the intracellular GSH level (Figure 3E, 3F), and reversed JAK/STAT3 signaling activation in response to cisplatin treatment (Figure 3G). The data suggests cisplatin induces activation of IL6/JAK/STAT3 signaling by reprogramming GSH metabolism.

### Inhibition of JAK/STAT3 alleviates cisplatin-induced apoptosis and ferroptosis and protects renal function against cisplatin nephrotoxicity

**Figure 4.**
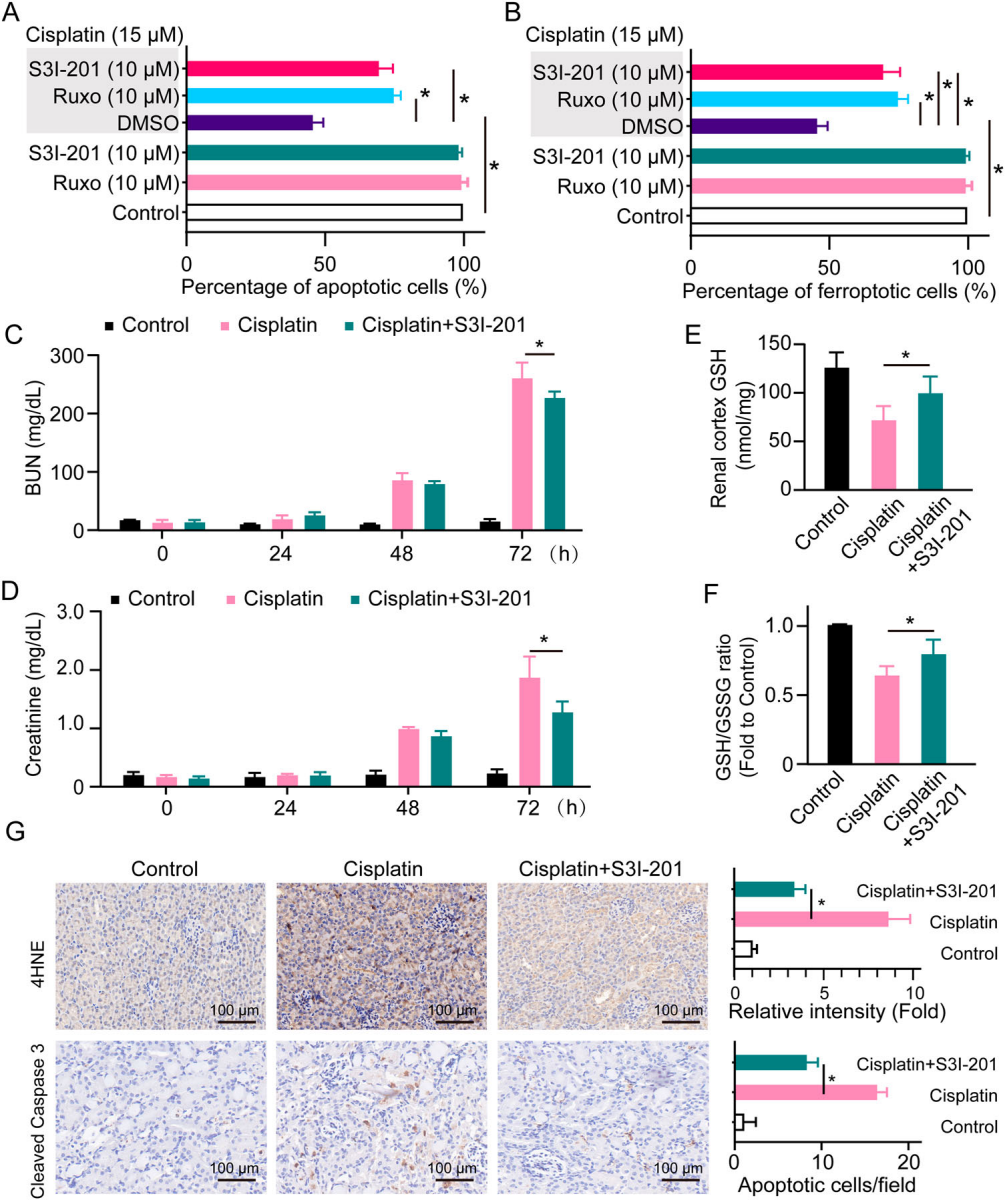
Inhibition of JAK/STAT3 protects against cisplatin nephrotoxicity. Cell apoptosis (A) and ferroptosis (B) were detected in HK2 cells treated with 15 μM of cisplatin in the presence or absence of 10 μM S3I-201 or 10 μM ruxolitinib for 24 h. Mice injected with cisplatin (30 mg/kg) or cisplatin (30 mg/kg) plus S3I-201 (5 mg/kg) or the same amount of PBS. The levels of BUN (C) and creatinine (D) were determined in serum collected from C57BL/6J mice at 0, 24, 48, and 72 h. The levels of GSH (E) and GSH/GSSG ratio (F) were determined in the renal cortex of C57BL/6J mice 72 h after cisplatin treatment. (G) IHC staining and quantification of cleaved caspase 3 and 4HNE on the kidneys from each group of mice. Statistical significance between the two groups as indicated was determined using an unpaired two-tailed Student’s t-test. Data are presented as mean ± S.E.M., *n* = 5, *p<0.05.

As the activation of JAK/STAT3 is induced by cisplatin, we investigated if targeting JAK/STAT3 could alleviate cisplatin-induced apoptosis and ferroptosis in renal tubular cells. We selected two JAK/STAT3 inhibitors including a preclinical chemical compound S3I-201 and a widely used clinical drug ruxolitinib. We found that S3I-201 and ruxolitinib markedly reduced the cytotoxic effects of cisplatin and reversed cisplatin-tin-induced apoptosis as well as ferroptosis in renal tubular cells (Figure 4A, 4B), indicating the potential therapeutic efficacy of ruxolitinib for cisplatin nephrotoxicity. We next investigated the effects of targeting JAK/STAT3 on cisplatin nephrotoxicity. The cisplatin-induced acute kidney injury was established following S3I-201 treatment. We found that S3I-201 could alleviate cisplatin-induced increased serum BUN and creatinine levels (Figure 4C, 4D), as well as promote the total GSH level and GSH/GSSG ratio in the renal cortex (Figure 4E, 4F). IHC staining for caspase-3 and 4HNE showed that cisplatin-tin-induced remarkable apoptosis and ferroptosis in the kidney were reversed by S3I-201 (Figure 4G), suggesting that targeting JAK/STAT3 protects renal function against cisplatin nephrotoxicity. The data showed that modulation of JAK/STAT3 alleviated cisplatin-induced apoptosis and ferroptosis and protects renal function against cisplatin nephrotoxicity.

### Baicalein protects renal function against cisplatin nephrotoxicity

**Figure 5.**
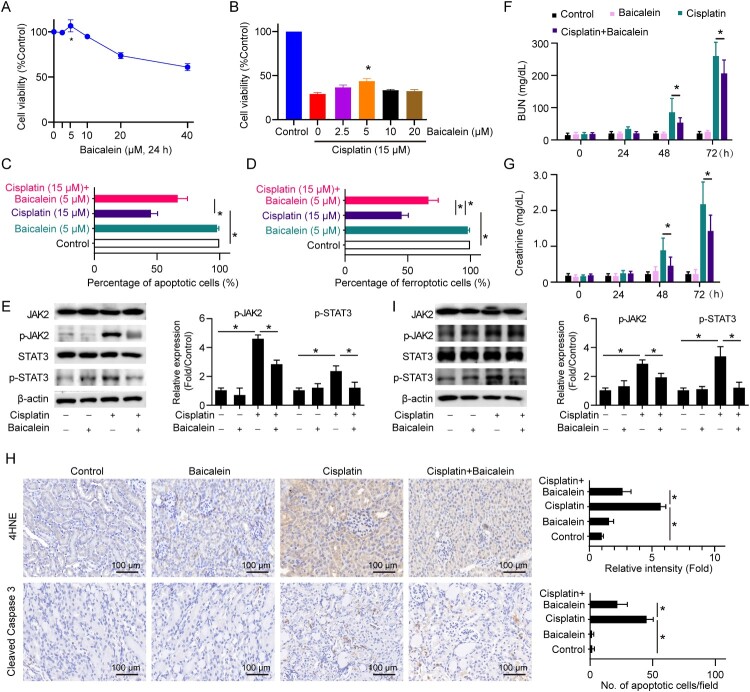
Baicalein protects renal function against cisplatin nephrotoxicity. (A) Cell viability was detected in HK2 cells treated with different doses (0-40 μM) of baicalein for 24 h. (B) Cell viability was detected in HK2 cells treated with 15 μM of cisplatin in the presence or absence of different doses (0-20 μM) of baicalein as indicated for 24 h. Cell apoptosis (C) and ferroptosis (D), expressions of the JAK/STAT signaling-associated proteins (E) were detected in HK2 cells treated with 15 μM of cisplatin in the presence or absence of 5 μM baicalein for 24 h. Mice were injected with cisplatin (30 mg/kg) or cisplatin (30 mg/kg) plus baicalein (10 mg/kg) or the same amount of PBS. BUN (F) and creatinine (G) levels were determined in serum collected from C57BL/6J mice at 0, 24, 48, and 72 h. (H) IHC staining and quantification of cleaved caspase 3 and 4HNE on the kidneys from each group of mice. (I) expressions of the JAK/STAT signaling-associated proteins (E) were detected in the kidney cortex from each group of mice. Statistical significance between the two groups as indicated was determined using an unpaired two-tailed Student’s t-test. Data are presented as mean ± S.E.M., *n* = 5, *p<0.05.

**Figure 6.**
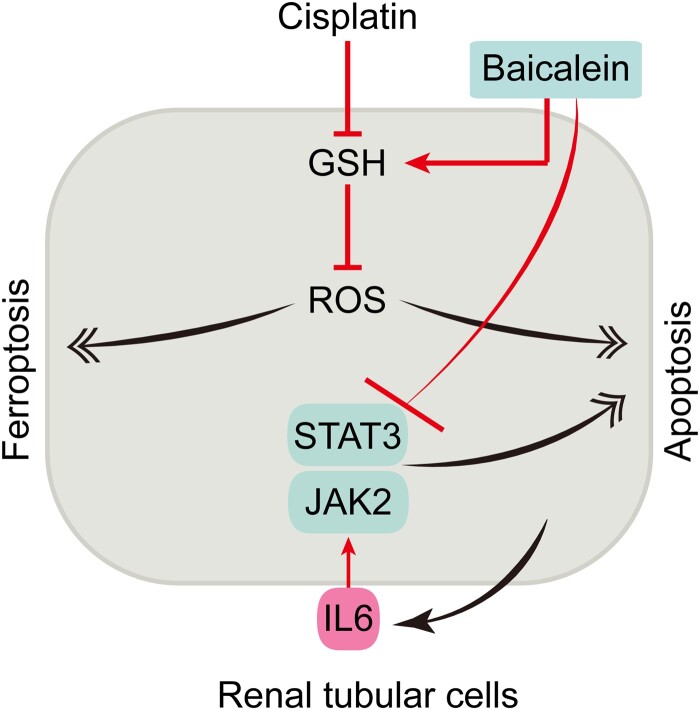
Schematic model for the mechanism of action under cisplatin nephrotoxicity. Cisplatin induces both apoptosis and ferroptosis in renal tubular cells by disrupting GSH metabolism leading to apoptosis and ferroptosis. Targeting JAK/STAT3 and/or GSH repletion using baicalein attenuates tubular cell apoptosis and ferroptosis and inhibits cisplatin nephrotoxicity.

A broad range of traditional herbs shows antioxidative activity and has the potential to mediate GSH metabolism [23]. The antioxidant baicalein has been reported to protect renal tubular cells from adenine-induced ferroptosis [24], and hypoxia-reoxygenation-induced apoptosis [25]. To advance our findings for further application broadly, we tested the effects of baicalein on the cell viability of renal tubular cells and the recovery of cisplatin-induced cell apoptosis and ferroptosis. We found a low dose of baicalein had minimal effects on cell viability with a sightly promoting impact at 5 μM. Whereas, higher doses (>10 μM) of baicalein showed remarkable cytotoxicity on renal tubular cells (Figure 5A). Consistently, baicalein significantly attenuated cisplatin-induced cytotoxicity with a better efficacy at 5 μM (Figure 5B). We next investigated the recovery of cisplatin-induced cell apoptosis and ferroptosis by using 5 μM of baicalein. We found baicalein significantly reversed cisplatin-induced renal tubular cell apoptosis and ferroptosis *ex vivo* (Figure 5C, 5D). Moreover, the activation of JAK/STAT3 induced by cisplatin was attenuated by baicalein treatment (Figure 5E). The functional study *in vivo* showed that baicalein significantly ameliorated kidney dysfunction and renal tubular cell apoptosis and ferroptosis caused by cisplatin *in vivo* (Figure 5F-5H). Furthermore, baicalein significantly repressed JAK/STAT3 in the cisplatin-caused mouse kidney *in vivo* (Figure 5I). Collectively, our data show that cisplatin induces both apoptosis and ferroptosis in renal tubular cells by disrupting GSH metabolism. GSH depletion impairs mitochondrial oxidation phosphorylation leading to mitochondria-mediated apoptosis and lipid oxidation-related ferroptosis. The inflammatory cytokine IL6 activates the downstream JAK/STAT3 signaling, which aggravates cell apoptosis and ferroptosis. Targeting JAK/STAT3 and/or GSH repletion using baicalein represents a promising strategy for combating cisplatin nephrotoxicity (Figure 6).

## Discussion

Cisplatin-induced acute kidney injury is manifested by the loss of proximal tubular cells and rapid decline of renal function, which eventually develops into kidney fibrosis and chronic kidney diseases (CKD) [26]. Cisplatin-induced proximal tubular cell death is executed through different types of machinery including apoptosis, ferroptosis, necro-sis, etc [15]. Current strategies attempting to rescue cisplatin-induced nephrotoxicity through ameliorating cell apoptosis, or ferroptosis elicit limited beneficial effects. Accordingly, uncovering the mechanism of action controlling both apoptosis and ferroptosis induced by cisplatin in renal tubular cells is highly necessary. Identification of specific targets or signaling that mediate cisplatin’s cytotoxicity will facilitate the development of novel therapies for CAKI.

The renal tubular cells represent the fundamental unit of the nephron that is responsible for normal renal function for absorption and filtration and thus are susceptible to different renal toxic substances [27]. As a widely used chemotherapeutic drug for cancer treatment, apart from inducing cancer cell apoptosis, cisplatin incurred both ferroptosis and apoptosis in normal renal tubular cells simultaneously which largely accounts for the mechanism of action of cisplatin’s nephrotoxicity. Therefore, the detailed molecular events under cisplatin-induced ferroptosis and apoptosis in renal tubular cells are necessary for cisplatin nephrotoxicity therapy. Lipid peroxidation is the hallmark of ferroptosis, which is regulated by GPX4 to protect cells from death triggered by diverse oxidative stress conditions [28]. We found that the reduction of GPX4 expression in response to cisplatin treatment supporting the ferroptosis existed on cisplatin-induced nephrotoxicity. By analyzing the enriched signaling pathways, we found that GSH metabolism is impaired in response to cisplatin. Amino acid metabolism is important to maintain renal cell homeostasis and cell function; disruption of metabolic homeostasis leads to cell death and tissue remolding in AKI [29]. GSH metabolism is one of the most important antioxidative metabolic pathways in mediating the intracellular redox reaction and oxidative phosphorylation (OXPHOS) in mitochondria. Dysregulated GSH metabolism will lead to imbalanced redox and accumulation of reactive oxygen species (ROS), which triggers lipid peroxidation-related cell ferroptosis and mitochondria-mediated intrinsic cell apoptosis [17,30]. In line with our findings, cisplatin-induced apoptosis, and ferroptosis in renal tubular cells through disrupting GSH metabolism, which is reversed by removing ROS. Our data showed that targeting GSH metabolism is a promising method to combat cisplatin-induced cisplatin and ferroptosis in renal tubular cells.

GSH metabolism was regulated in a precise way and associated with multiple cellular events [16,19]. In response to stimuli, GSH metabolism reprogramming inter-playing with other signaling pathways involves the development and progression of different diseases. It reported that activation of JAK/STAT3 signaling was involved in cisplatin-induced acute kidney injury [31]. In consistent, we found JAK/STAT3 signaling was activated in renal tubular cells in response to cisplatin, suggesting a potential interplay between the GSH metabolism and JAK/STAT3 signaling in mediating cisplatin-tin-induced cell apoptosis and ferroptosis in renal tubular cells. A previous study demonstrated that JAK/STAT3 is activated by IL6 and controlled by GSH metabolism in cardiomyocytes [22]. This report is consistent with our finding that cisplatin-tin-induced cells apoptosis with an increased inflammatory cytokine IL6, which triggers JAK/STAT3 activation, aggravating cell apoptosis and ferroptosis. Therefore, targeting JAK/STAT3 signaling represents a promising way to stop cisplatin-induced nephrotoxicity. In our study, we used two JAK/STAT3 inhibitors S3I-201 and ruxolitinib to test their therapeutic efficacy ex vivo. We found inhibition of JAK/STAT3 reversed cisplatin-induced cell apoptosis and ferroptosis in renal tubular cells. in addition, the potential preclinical therapeutic effects were also investigated using S3I-201 to treat CAKI mice and found that renal function was alleviated by inhibition of JAK/STAT3. Ruxininib is an FDA-approved orally delivered drug for treating cancer. Considering the different ways of drug delivery, the similar therapeutic effects on CAKI for ruxininib and S3I-201 remain undetermined and need to be further investigated.

Many traditional natural herbals have attracted considerable attention to be used for AKI treatment due to their advantages such as better accessibility and lower toxicity [32]. Baicalein, a flavone and an active ingredient in the traditional herbal Huang Qin, has been used to treat various types of disease through its multiple pharmacological effects such as anti-inflammatory, and antioxidant [33]. The antioxidative activity of baicalein has been reported to protect renal tubular cells from adenine-induced ferroptosis [24], and hypoxia-reoxygenation-induced apoptosis [25], showing the potential to mediate GSH metabolism. We found baicalein showed protective effects on renal tubular cells at a lower dose but showed cytotoxicity at a higher dose. A low dose of baicalein effectively protected cisplatin-induced ferroptosis and apoptosis ex vivo and alleviated cisplatin-induced renal dysfunction in vivo. In accordance, the application of baicalein has achieved favorable outcomes on renal function. It broadens our findings to a higher level that explores more drugs with antioxidative activities for AKI therapy under cisplatin induction.

In conclusion, we have demonstrated that cisplatin-induced nephrotoxicity was displayed by inducing renal tubular cells in both apoptosis and ferroptosis and found that GSH metabolism imbalance and JAK/STAT3 signaling activation was involved in this process. Cisplatin-induced GSH depletion impaired mitochondria-mediated apoptosis and lipid oxidation-related ferroptosis in renal tubular cells. The inflammatory cytokine IL6 produced from apoptotic cells activated the downstream JAK/STAT3 signaling pathway, which further aggravates cell apoptosis and ferroptosis. Baicalein showed great potency for reversing cisplatin-induced renal tubular cell apoptosis and ferroptosis ex vivo and cisplatin-caused kidney dysfunction in vivo by modulating JAK/STAT3 signaling. Inhibition of JAK/STAT3 using baicalein represents a promising strategy for combating cisplatin nephrotoxicity.

## Author contributions

Conception and design, J.L. X.M.Y.; acquisition and analysis of data, X.Q.D., L.K.C., J.L., and X.C.; writing, review, and/or revision of the manuscript, J.L., X.M.Y., S.W.H., and C.-H.C.; administrative, technical, or material support, Q.W.X., Y.M.M., and Y.L.B.; study super-vision, J.L. X.M.Y. All authors have read and agreed to the published version of the manuscript.

## Data Availability

The data presented in this study are available upon request from the corresponding author.
